# Kyoto probe-1 reveals phenotypic differences between mouse ES cells and iTS-P cells

**DOI:** 10.1038/s41598-020-75016-6

**Published:** 2020-10-22

**Authors:** Chika Miyagi-Shiohira, Issei Saitoh, Masami Watanabe, Hirofumi Noguchi

**Affiliations:** 1 Department of Regenerative Medicine, Graduate School of Medicine, University of the Ryukyus, 207 Uehara, Nishihara, Okinawa 903-0215 Japan; 2 Division of Pediatric Dentistry, Graduate School of Medical and Dental Science, Niigata University, Niigata, 951-8514 Japan; 3 Department of Urology, Okayama University Graduate School of Medicine, Dentistry and Pharmaceutical Sciences, Okayama, 700-8558 Japan

**Keywords:** Cell biology, Stem cells

## Abstract

Kyoto probe 1 (KP-1) rapidly distinguishes between human ES/iPS (hES/iPS) cells and their differentiated cells. Recently, we generated induced tissue-specific stem cells from pancreas (iTS-P cells) using reprogramming factors and tissue-specific selection. The iTS-P cells have self-renewal potential, and subcutaneously transplanting them into immunodeficient mice did not generate teratomas. In this study, we applied KP-1 to analyze mouse ES (mES) cells and mouse iTS-P (miTS-P) cells. KP-1 completely stained mES cells in colonies, but only miTS-P cells at the edge of a colony. This difference was caused by cell type-specific expression of different ABC transporters. These finding suggest that KP-1 will be useful for distinguishing between iPS and iTS-P cells.

## Introduction

Pluripotent stem (PS) cells hold great promise for the development of stem cell therapies to treat diverse human diseases^1–12^. However, numerous limitations hinder the clinical application of embryonic stem (ES) cells and induced pluripotent stem (iPS) cells, including the tumorigenic risk of transplanted undifferentiated cells^13–17^. The Kyoto probe 1 (KP-1), which selectively labels human PS cells^18^, was developed as a selection tool. KP-1 rapidly distinguishes between human ES/iPS (hES/hiPS) cells and differentiated cells. Further, KP-1 is a stable, chemically defined small molecule that offers the advantages of ease of use and economy. These properties are critically important for mitigating the risk of stem cell therapy-induced tumorigenesis as well as for conducting basic stem cell research^18^. The unique selectivity of KP-1 is primarily explained by the distinct expression patterns of ATP-binding cassette (ABC) transporters by hiPS cells and differentiated cells. ABCB1 (also known as MDR1, PGY1) and ABCG2 (also known as ABCP, BCRP) mediate the efflux of KP-1, which is repressed in hiPS and hES cells. In contrast, differentiated human cells express ABCB1 and ABCG2^18,19^. The drug efflux systems mediated by ABC transporters in human cells are well characterized^20–24^, in contrast to those of mouse cells.

Induced tissue-specific stem (iTS) cells can be generated by transient overexpression of the reprograming factors Oct4, Klf4, Sox2, Lin28, p53 shRNA, L-Myc and/or c-Myc followed by tissue-specific selection^25–27^. iTS cells are generated as well using the unique synthetic, self-replicating VEE-RF RNA replicon that encodes OCT4, KLF4, SOX2, and GLIS1^28,29^. Transfection of mouse pancreatic tissue with VEE-RF RNA efficiently generates pancreatic iTS cells (iTS-P cells), which express markers characteristic of endodermal and pancreatic progenitors. Such cells differentiate into insulin-producing cells more efficiently than ES cells. iTS-P cells self-renew, and subcutaneous transplantation of these cells into immunodeficient mice does not generate teratomas^28^.

Here we show that KP-1 distinguishes between mouse ES/iPS (mES/miPS) cells and mouse iTS-P (miTS-P) cells because of differential expression of ABC transporters.

## Results

### Characteristics of mES and miTS-P cells

We generated mouse iTS cells from 24-week-old donor pancreata transfected with the self-replicating VEE-RF RNA replicon that expresses the reprogramming factors OCT4, KLF4, SOX2, and GLIS1^28,29^. The miTS-P cells exhibited a “cobblestone-like” morphology, similar to that of mouse pancreatic stem cells (Supplementary Fig. 1A), which we established from pancreata of young donors^30^. We analyzed the levels of markers of pluripotency and endoderm/pancreatic progenitors expressed by mES and miTS-P cells. The levels of the pluripotency markers Nanog, Sox2, Oct3/4, Lin28a, Nodal, and Rex1 expressed by miTS-P cells were significantly lower compared with those expressed by mES cells. The expression of endodermal marker genes (Hnf1β, 4α, Foxa2, Sox17and CD133) was detected in miTS-P cells but not in mES cells (Supplementary Fig. 1B). Subcutaneous transplantation of mES cells, but not miTS-P cells, into immunodeficient mice resulted in teratoma formation (Supplementary Fig. 1C).

**Figure 1:**
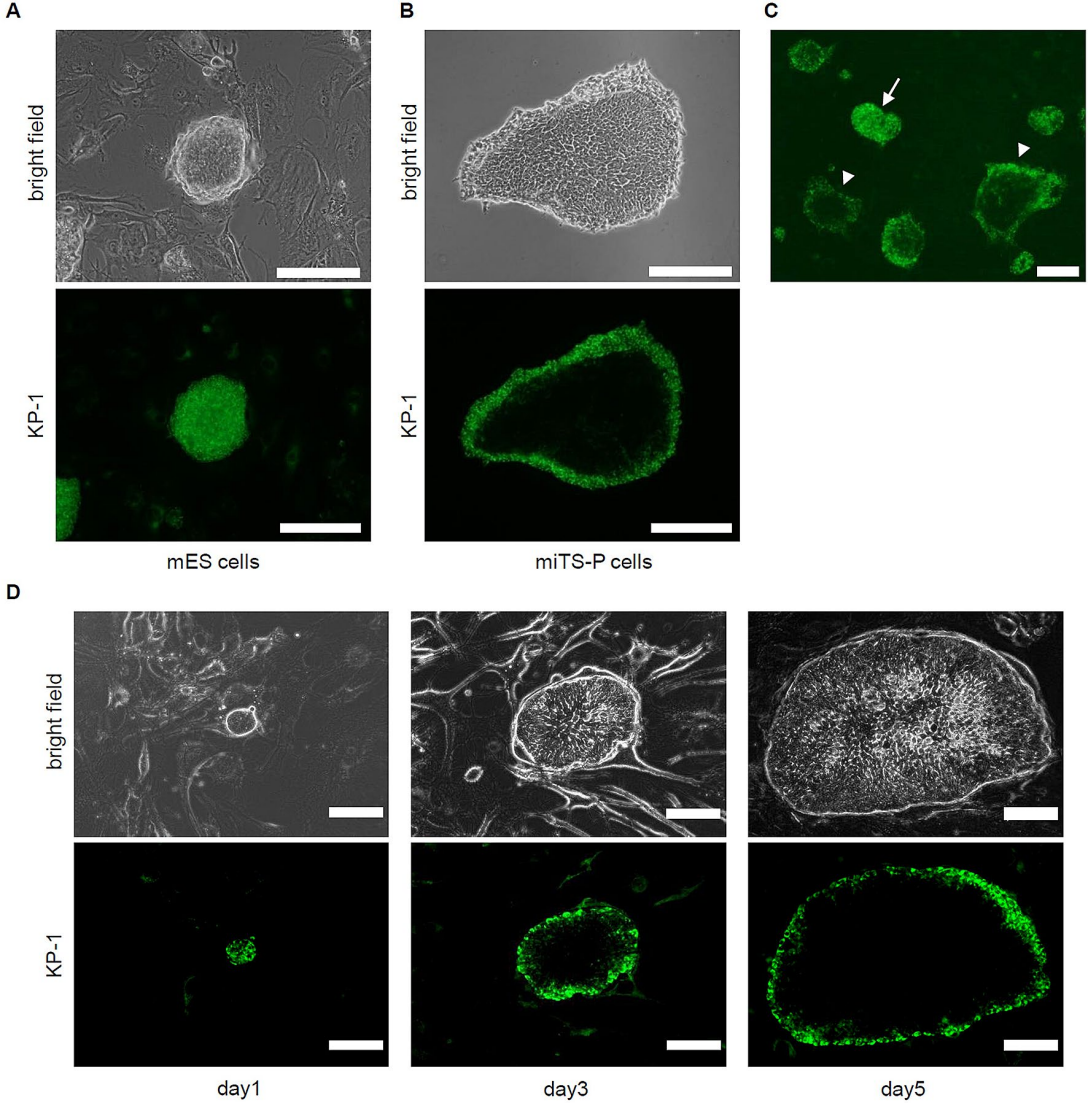
KP-1 treatment of mES, miTS-P, and mixtures of miPS/miTS-P cells. (**A**) mES cells were added (2 × 10^5^ cells per well) to a six-well plate and cultured for 5 days, treated with 2 µM KP-1 for 3 h at 37 °C, rinsed with PBS, and images were acquired using a fluorescence microscope. Scale bar = 200 µm. (**B**) miTS-P cells were added (2 × 10^5^ cells per well) to a six-well plate and cultured for 5 days, treated with 2 µM KP-1 for 3 h at 37 °C, rinsed with PBS. Scale bar = 200 µm. (**C**) A mixture of miPS/miTS-P cells was added (2 × 10^5^ cells per well) to a six-well plate and cultured for 5 days, treated with 2 µM KP-1 for 3 h at 37 °C, and rinsed with PBS. Scale bar = 200 µm. arrow; miPS cells, arrow head; miTS-P cells (**D**) Time-course of miTS-P cells treated with KP-1. miTS-P cells were added (2 × 10^5^ cells/well) to a six well plate. miTS-P cells were cultured from days 1 to 3 and 5 and then treated with 2 µM KP-1 for 3 h at 37 °C Cells were rinsed with PBS, and. Scale bar = 200 µm.

### KP-1 analysis of mES, miTS-P, and miPS cells

mES, miTS-P, or a mixture of miPS/miTS-P cells were incubated with KP-1 on day 5 after passage. All mES cells in colonies were stained by KP-1 as are hES/iPS cells as previously reported^18^ (Fig. 1A). However, KP-1 only stained border cells of the miTS-P colonies (Fig. 1B). Some colonies derived from one culture of miPS/miTS-P cells stained similarly to ES cells, which we considered miPS cells, while border cells of other colonies were stained by KP-1, which we considered miTS-P cells (Fig. 1C). We next investigated the characteristics of miTS-P cells treated with KP-1 on days 1, 3, and 5. On day 1, all miTS-P cells were stained by KP-1. However only border cells of the colonies were stained on days 3 and 5 (Fig. 1D), suggesting that the phenotype of miTS-P cells in the center of the colony changed during cell division.

### Changes in KP-1 staining of mES and miTS-P cells before and after cell passage

**Figure 2:**
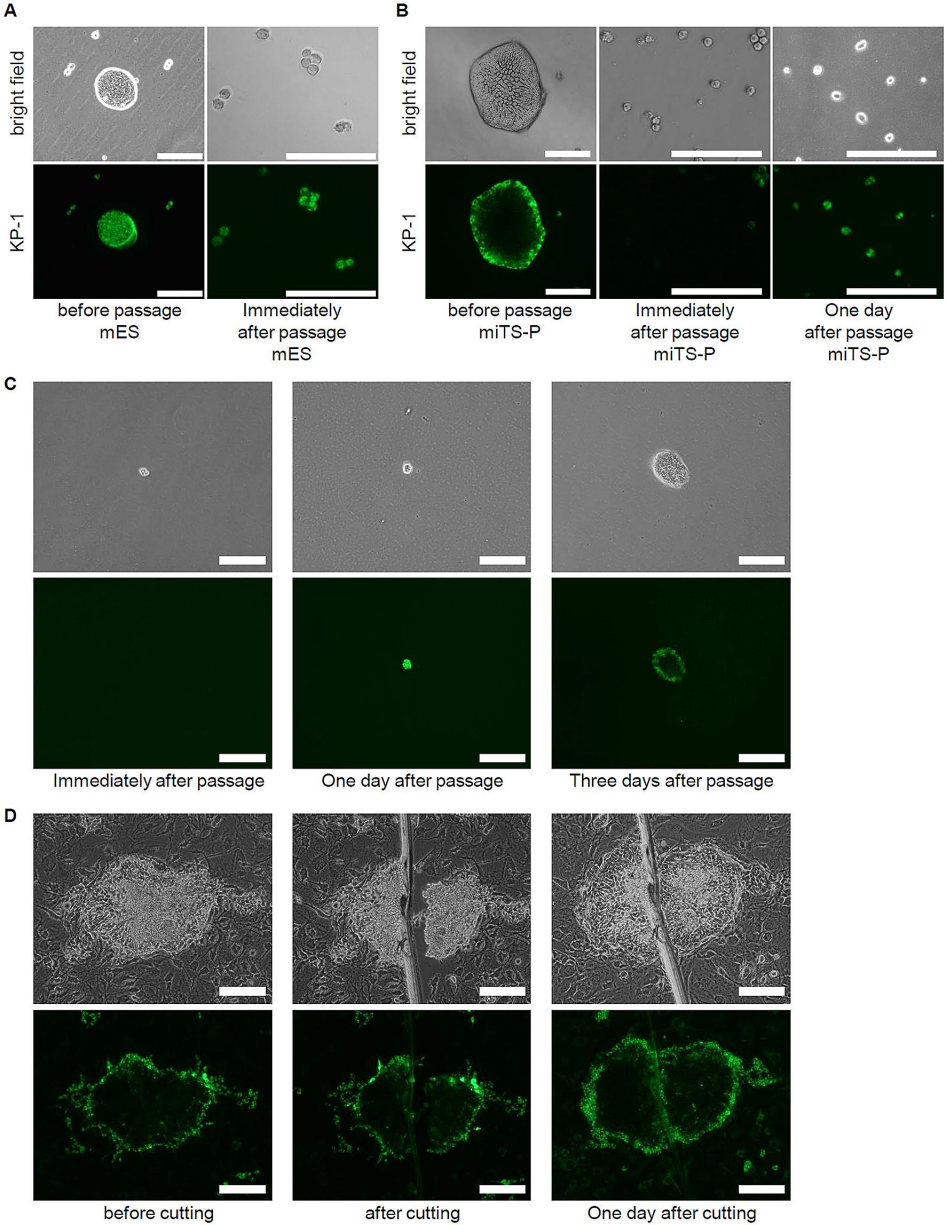
KP-1 staining of mES and miTS-P cells after passage. (**A**) mES cells were treated with 2 µM KP-1 for 3 h at 37 °C and rinsed with PBS. The cells were then passaged. (**B**) miTS-P cells were treated with 2 µM KP-1 for 3 h at 37 °C and rinsed with PBS. The cells were then passaged and cultured for one day, re-treated with 2 µM KP-1 for 3 h at 37 °C, and rinsed with PBS. Scale bar = 200 µm. (**C**) miTS-P cells were treated with 2 µM KP-1 for 3 h at 37 °C and rinsed with PBS. The cells were then passaged. A single KP-1-negative cell was sorted and replated. The cell was cultured for 1–3 days, treated with 2 µM KP-1 for 3 h at 37 °C, then rinsed with PBS. (**D**) A colony formed by miTS-P cells was treated with 2 µM KP-1 for 3 h at 37 °C and rinsed with PBS. The colonies were cut with a knife and cultured for one day. The cells were re-treated with 2 µM KP-1 for 3 h at 37 °C, then rinsed with PBS.

We investigated whether KP-1-negative cells present in the center of miTS-P colonies had a differentiated or stem-cell phenotype. To exclude contamination by feeder cells, mES and miTS-P cells were cultured in a feeder-free, laminin-coated flask. The mES and miTS-P cells were stained before passage. After cell passage, almost all mES cells were KP-1-positive (Fig. 2A). In contrast, approximately 20% of trypsinized miTS-P cells were KP-1-positive while the remaining percentage were KP-1-negative immediately after cell passage (Fig. 2B). One day after cell passage, 90% of cells were KP-1-positive (Fig. 2B). These cells self-renewed and exhibited a morphology similar to that of miTS-P cells 5 days after passage. To determine whether KP-1-negative cells became KP-1-positive after one day in culture and formed a colony, single KP-1-negative cell was sorted and replated. The KP-1-negative cells on day 0 became KP-1-positive on day 1 and formed colonies on day 3 (Fig. 2C). To determine whether KP-1-negative central cells became KP-1-positive, we cut a colony of miTS-P cells on day 5 and cultured the colony for one day. KP-1-negative cells in the center of the miTS-P colony became KP-1-positive in one day (Fig. 2D). These data suggest that KP-1-negative cells converted to KP-1-positive cells after passage and form colonies.

### Flow cytometric analysis of mES and miTS-P cells treated with KP-1

**Figure 3:**
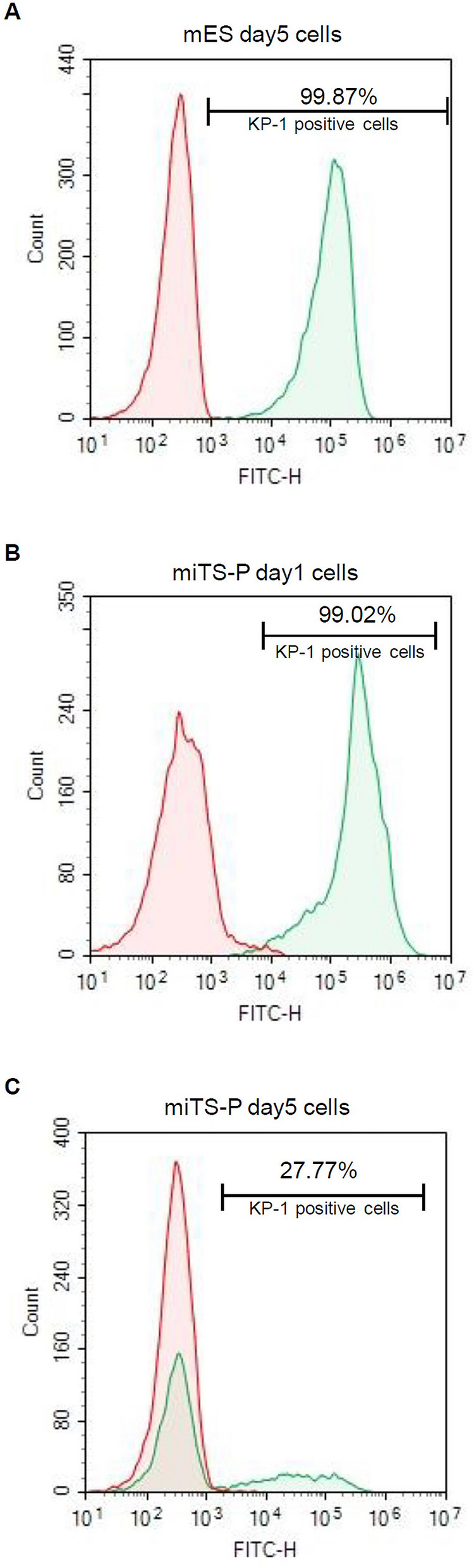
Flow cytometric analysis of mES and miTS-P cells with KP-1 staining. After treating mES and miTS-P cells with KP-1 and dissociating them with 0.25% trypsin–EDTA, they were counted using a Novocyte Flow Cytometer. (**A**) mES cells five days after cell passage. (**B**) miTS-P cells one day after cell passage. (**C**) miTS-P cells five day after cell passage. mES or miTS-P cells not treated with KP-1 were used as negative control.

We performed flow cytometry to determine the abundances of KP-1-positive and KP-1-negative mES and miTS-P cells after passage. On day 5 after passage, 99.87% of mES cells were KP-1-positive (Fig. 3A). On days 1 and 5 after passage, 99.02% and 27.77% of miTS-P cells were KP-1-positive, respectively (Fig. 3B,C).

### Microarray analysis of the expression of genes encoding ABC transporters by mES and miTS-P cells

ABC transporters are components of systems that mediate drug efflux^31^. We therefore compared the expression profiles of genes encoding ABC transporters in mES cells and miTS-P cells. It has been reported that ABCB1 and ABCG2 mediate the efflux of KP-1, which is repressed in hES/hiPS cells^19,32^. However, microarray analysis detected the expression of Abcb1 and Abcg2 in mES and miTS-P cells (Table 1). These data indicate that Abcb1 and Abcg2 were unlikely to mediate the efflux of KP-1 in mouse cells. According to the microarray data, we selected for further analysis Abcc5 (also known as MRP5), Abcc10 (also known as MRP7), Abcc12 (also known as MRP9), and Abca2 (also known as ABC2) as ABC transporters that potentially mediated the efflux of KP-1, because these genes were expressed by miTS-P cells but not by mES cells (Table 1).

**Table 1:** Microarray analysis of mES and miTS-P ABC transporters.

Gene symbol	ES	Detection	iTS-P	Detection	Representative public ID	Chromosomal location
Abca1	7167.403	P	452.154	P	BB144704	chr4 28.57 cM|4 A5-B3
Abca1	168.4587	P	15.51394	A	BB144704	chr4 28.57 cM|4 A5-B3
Abca1	481.5598	P	35.337	A	BB144704	chr4 28.57 cM|4 A5-B3
Abca12	40.54979	A	68.8113	A	AK014713	chr1|1 C3
Abca13	13.67649	A	24.42159	A	BB277120	chr11|11 A2
Abca13	6.920056	A	58.58181	A	BB503961	chr11|11 A2
Abca14	26.56111	A	18.67748	P	AK015996	chr7|7 F2
Abca16	4.930875	A	1.072923	A	AV257295	chr7|7 F2
**Abca2**	**81.51833**	**A**	**189.0628**	**P**	**NM_007379**	**chr2 17.25 cM|2 A2-B**
Abca3	24.32789	A	44.39275	A	BC006932	chr17|17 A3.3
Abca3	209.3091	P	425.7065	P	AK007703	chr17|17 A3.3
Abca3	602.3256	P	981.3214	P	AK007703	chr17|17 A3.3
Abca4	25.66407	A	33.61237	A	NM_007378	chr3 G1|3 52.94 cM
Abca5	1266.377	P	867.3645	P	BM937648	chr11|11 E1
Abca5	49.24316	P	112.044	P	BB128256	chr11|11 E1
Abca6	24.94242	M	29.81462	P	AK018242	chr11|11 E1
Abca7	255.0093	P	602.5334	P	NM_013850	chr10 B4-C1|10 39.72 cM
Abca8a	211.3128	P	52.51521	P	BC026496	chr11|11 E1
Abca8b	131.5535	P	126.3798	P	AF213393	chr11 E1|11 72.88 cM
Abca9	738.4904	P	55.25328	A	AW046072	chr11|11 E1
Abca9	131.8562	M	36.57425	A	BB497208	chr11|11 E1
Abcb10	229.8845	P	232.7289	P	AV382118	chr8|8 E2
Abcb10	467.127	P	629.3079	P	AV382118	chr8|8 E2
Abcb10	49.59097	A	59.27524	A	AK011569	chr8|8 E2
Abcb11	6.996332	A	7.74302	A	NM_021022	chr2 C2|2 39.69 cM
**Abcb1a**	**151.0668**	**P**	**548.7768**	**P**	**M30697**	**chr5 A1|5 3.43 cM**
**Abcb1a**	**164.1348**	**P**	**462.7107**	**P**	**M30697**	**chr5 A1|5 3.43 cM**
**Abcb1b**	**63.46278**	**A**	**29.89989**	**A**	**BB399117**	**chr5 A1|5 3.43 cM**
**Abcb1b**	**2339.168**	**P**	**545.6501**	**P**	**NM_011075**	**chr5 A1|5 3.43 cM**
Abcb4	100.7659	P	63.12254	P	NM_008830	chr5 A1|5 3.43 cM
Abcb6	224.6298	P	337.4362	P	NM_023732	chr1|1 C3
Abcb7	773.6096	P	1576.549	P	BM119407	chrX 46.58 cM|X C-D
Abcb7	72.22068	P	127.851	P	AW537380	chrX 46.58 cM|X C-D
Abcb7	123.7106	P	217.614	P	U43892	chrX 46.58 cM|X C-D
Abcb8	147.9785	P	196.8055	P	BC015301	chr5|5 A3
Abcb8	409.8591	P	497.6263	P	NM_029020	chr5|5 A3
Abcb9	21.35689	A	80.1112	A	AB045382	chr5|5 F
Abcb9	59.97397	A	33.86317	A	AB045382	chr5|5 F
Abcb9	30.21912	A	2.095512	A	AK020749	chr5|5 F
Abcc1	433.2639	P	445.0964	P	NM_008576	chr16|16 A1
Abcc1	1501.148	P	1565.688	P	BG071908	chr16|16 A1
Abcc10	87.34413	A	15.82719	A	AF417121	chr17|17 C
**Abcc10**	**7.188884**	**A**	**46.77325**	**M**	**BB079952**	**chr17|17 C**
Abcc12	26.63185	A	6.384811	A	AV277642	chr8 42.06 cM|8 D3
**Abcc12**	**28.932**	**A**	**43.66473**	**P**	**BB013432**	**chr8 42.06 cM|8 D3**
Abcc2	9.587849	A	17.67956	A	NM_013806	chr19 C3|19 36.67 cM
Abcc3	226.5444	P	1339.783	P	AK006128	chr11|11 D
Abcc4	510.4953	P	3579.692	P	BB291885	chr14|14 E4
Abcc5	482.5937	P	436.5486	P	AV150520	chr16 A3|16 12.41 cM
Abcc5	76.01572	A	257.1242	A	BB138279	chr16 A3|16 12.41 cM
Abcc5	152.1698	P	302.6777	P	AF213387	chr16 A3|16 12.41 cM
**Abcc5**	**188.2284**	**A**	**386.6925**	**P**	**AV150520**	**chr16 A3|16 12.41 cM**
Abcc5	674.2209	M	767.2522	M	AV150520	chr16 A3|16 12.41 cM
Abcc5	417.2568	P	1094.376	P	BB436535	chr16 A3|16 12.41 cM
Abcc5	87.29051	P	175.5081	P	BB436535	chr16 A3|16 12.41 cM
Abcc5	9.567561	A	40.53646	A	AW456891	chr16 A3|16 12.41 cM
Abcc5	166.8643	P	562.0101	P	BB794846	chr16 A3|16 12.41 cM
Abcc6	6.349689	A	11.77285	A	NM_018795	chr7 29.64 cM|7 B3
Abcc8	26.00531	A	30.13037	A	BB515948	chr7 B4|7 29.66 cM
Abcc8	2.274869	A	40.46421	A	BF466569	chr7 B4|7 29.66 cM
Abcc9	9.117372	A	4.791087	A	BG791642	chr6 G2|6 74.35 cM
Abcc9	4.477273	A	3.426716	A	BG791642	chr6 G2|6 74.35 cM
Abcc9	6.296571	A	11.45889	A	NM_021043	chr6 G2|6 74.35 cM
Abcd1	313.9412	P	211.6199	P	BC011273	chrX 37.39 cM|X B
Abcd2	97.63587	P	19.23027	A	BB197269	chr15|15 E–F
Abcd2	97.96976	P	3.253005	A	NM_011994	chr15|15 E–F
Abcd2	24.26188	A	1.83638	A	AW456685	chr15|15 E–F
Abcd2	20.05282	A	9.718541	A	BB253618	chr15|15 E–F
Abcd3	3029.919	P	4008.688	P	BC009119	chr3 52.94 cM|3 G-H1
Abcd3	16.2971	A	6.350235	A	BB042134	chr3 52.94 cM|3 G-H1
Abcd4	166.7678	A	150.9212	A	AF213384	chr12 D1|12 39.3 cM
Abcd4	612.8154	P	423.0568	P	NM_008992	chr12 D1|12 39.3 cM
Abce1	4549.572	P	4548.196	P	NM_015751	chr8|8 C
Abce1	2483.66	P	2654.57	P	NM_015751	chr8|8 C
Abcf1	3391.364	P	2291.147	P	AA408356	chr17 B1|17 18.8 cM
Abcf1	135.7706	A	106.873	A	AA408356	chr17 B1|17 18.8 cM
Abcf1	992.0746	P	578.2582	P	BF236176	chr17 B1|17 18.8 cM
Abcf1	110.1304	P	76.10096	A	BF236176	chr17 B1|17 18.8 cM
Abcf1	1166.962	P	772.4178	P	AV309591	chr17 B1|17 18.8 cM
Abcf1	2689.757	P	1557.282	P	AV309591	chr17 B1|17 18.8 cM
Abcf2	153.7137	P	153.3369	P	BF143629	chr5|5 A3
Abcf2	809.0536	P	590.3427	P	BC003300	chr5|5 A3
Abcf3	64.25156	P	111.0832	P	AI552141	chr16 A3|16 12.46 cM
Abcf3	119.9229	P	204.8395	P	AI552141	chr16 A3|16 12.46 cM
Abcf3	508.7837	P	704.9908	P	AI552141	chr16 A3|16 12.46 cM
Abcg1	91.01067	A	39.34751	A	AW413978	chr17|17
Abcg1	6.707681	A	7.219179	A	BQ176322	chr17|17
**Abcg2**	**4221.282**	**P**	**1210.672**	**P**	**NM_011920**	**chr6 B3|6 27.82 cM**
Abcg3	5.365516	A	0.8682888	A	NM_030239	chr5 E5|5 50.68 cM
Abcg3	40.75346	A	43.58197	A	NM_030239	chr5 E5|5 50.68 cM
Abcg4	17.62659	A	22.99714	A	AY040865	chr9|9 A5.3
Abcg4	195.5778	A	146.0644	P	BC026477	chr9|9 A5.3
Abcg5	14.60767	A	38.49642	A	NM_031884	chr17 E4|17 55.02 cM
Abcg8	169.5168	P	141.6871	M	AF324495	chr17 E4|17 55.02 cM

### Quantitative reverse transcription-polymerase chain reaction (RT-qPCR) analysis of selected genes encoding ABC transporters in mES and miTS-P cells

**Figure 4:**
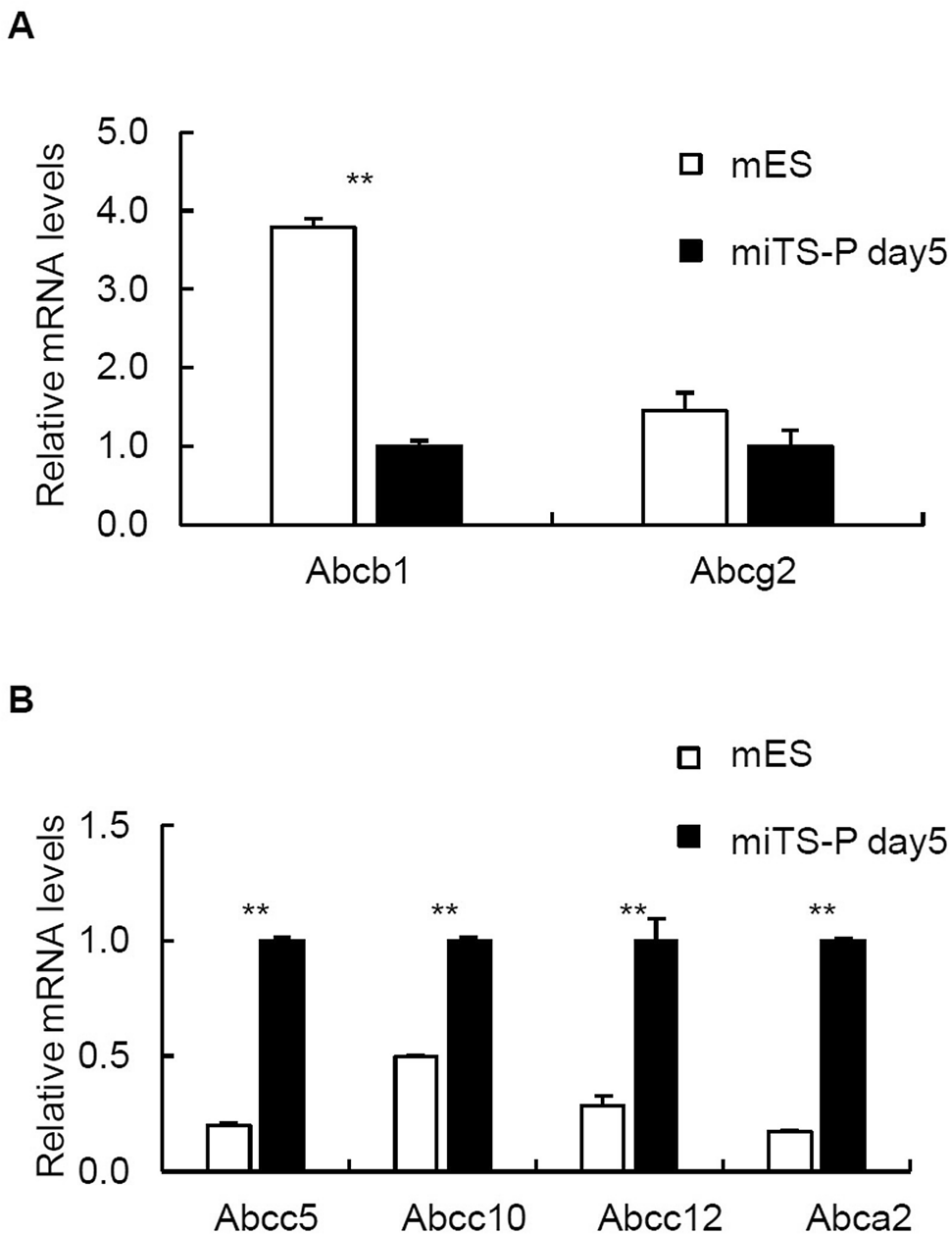
Expression profiles of ABC transporters of mES and miTS-P cells. (**A**) RT-qPCR analysis of Abcb1 and Abcg2 expression, mean ± SE. (n = 3). (**B**) RT-qPCR analysis of Abcc5, Abcc10, Abcc12, and Abca2 expression. Mean ± SE. (n = 3). ** *p* < 0.01.

We determined the levels of mRNAs encoding the ABC transporters Abcb1, Abcg2, Abcc5, Abcc10, Abcc12, and Abca2 in mES and miTS-P cells. Abcb1 and Abcg2 were expressed at significantly higher levels by mES cells compared with those expressed by miTS-P cells (Fig. 4A). These data suggest that Abcb1 and Abcg2 were unlikely to mediate the efflux of KP-1 from mES cells. In contrast, the levels of Abcc5, Abcc10, Abcc12, and Abca2 mRNAs in iTS-P cells were significantly higher compared with those in mES cells (Fig. 4B). These data suggest that at least one of these ABC transporters mediated the efflux of KP-1 from miTS-P cells.

### Analysis of KP-1 staining of miTS-P cells treated with inhibitors of ABC transporters

**Figure 5:**
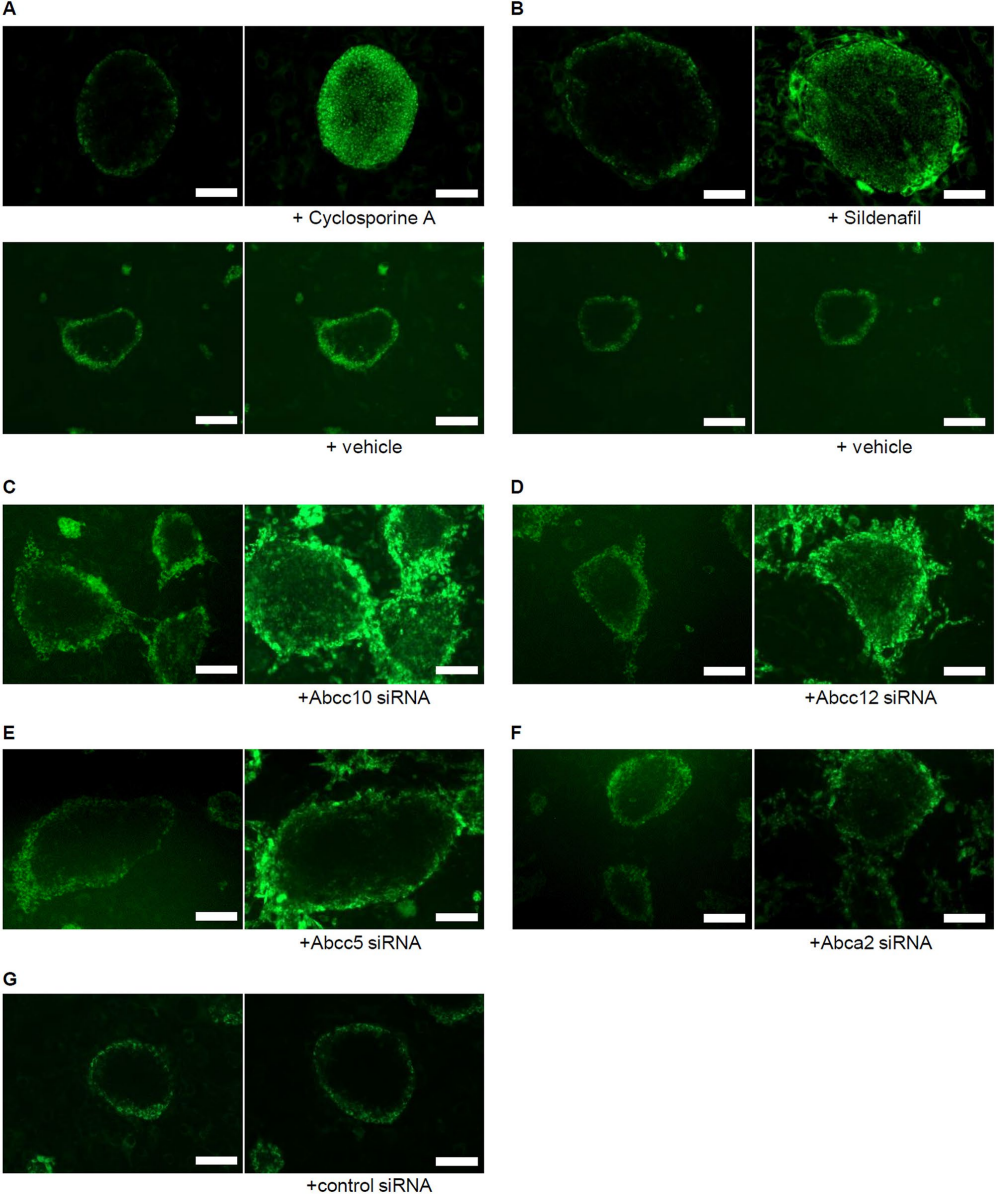
Effects of inhibitors of ABC transporters on KP-1 staining. (**A**) Cyclosporine A treatment. miTS-P cells 5 days after passage were treated with 2 µM KP-1 for 3 h at 37 °C. The cells were then cultured with the Abcc10 inhibitor cyclosporine A (5 µM) for 2 h, then treated with 2 µM KP-1 for 3 h at 37 °C. (**B**) Sildenafil treatment. miTS-P cells 5 days after cell passage were treated with 2 µM KP-1 for 3 h at 37 °C. The cells were then cultured with the Abcc5 inhibitor sildenafil (50 µM) for 2 h and then treated with 2 µM KP-1 for 3 h at 37 °C. (**C**–**G**) Treatment with siRNAs specific for cognate ABC transporters. miTS-P cells 5 days after passage were treated with 2 µM KP-1 for 3 h at 37 °C. The cells were then transfected with each siRNA of ABC transporter and cultured for 24 h, then treated with 2 µM KP-1 for 3 h at 37 °C. (**C**) Treatment with Abcc10-siRNA. (**D**) Treatment with Abcc12-siRNA. (**E**) Treatment with Abcc5-siRNA. (**F**) Treatment with Abca2-siRNA. (**G**) Treatment with control siRNA. Scale bar = 200 µm.

Sildenafil inhibits Abcc5^33,34^, and cyclosporine A (CsA) inhibits Abcc10^18,35^. KP-1 stained the border cells of the miTS-P colonies. After treatment with cyclosporine A, KP-1 stained all miTS-P cells (Fig. 5A), but only weakly after treatment with sildenafil (Fig. 5B). These data suggest that Abcc10 participated in mediating the efflux of KP-1 from miTS-P cells.

Next, when we used siRNAs specific for ABC transporters to understand their role in KP-1 selectivity (Supplemental Fig. 2), we found that KP-1 stained miTS-P cells after treatment with Abcc10-siRNA (Fig. 5C) or Abcc 12 siRNA (Fig. 5D). In contrast, the central cells of miTS-P colonies were weakly stained by KP-1 after treatment with Abcc5-siRNA (Fig. 5E), and central cells of miTS-P colonies were not stained by KP-1 after treatment with Abca2-siRNA (Fig. 5F) or control-siRNA (Fig. 5G).

**Figure 6:**
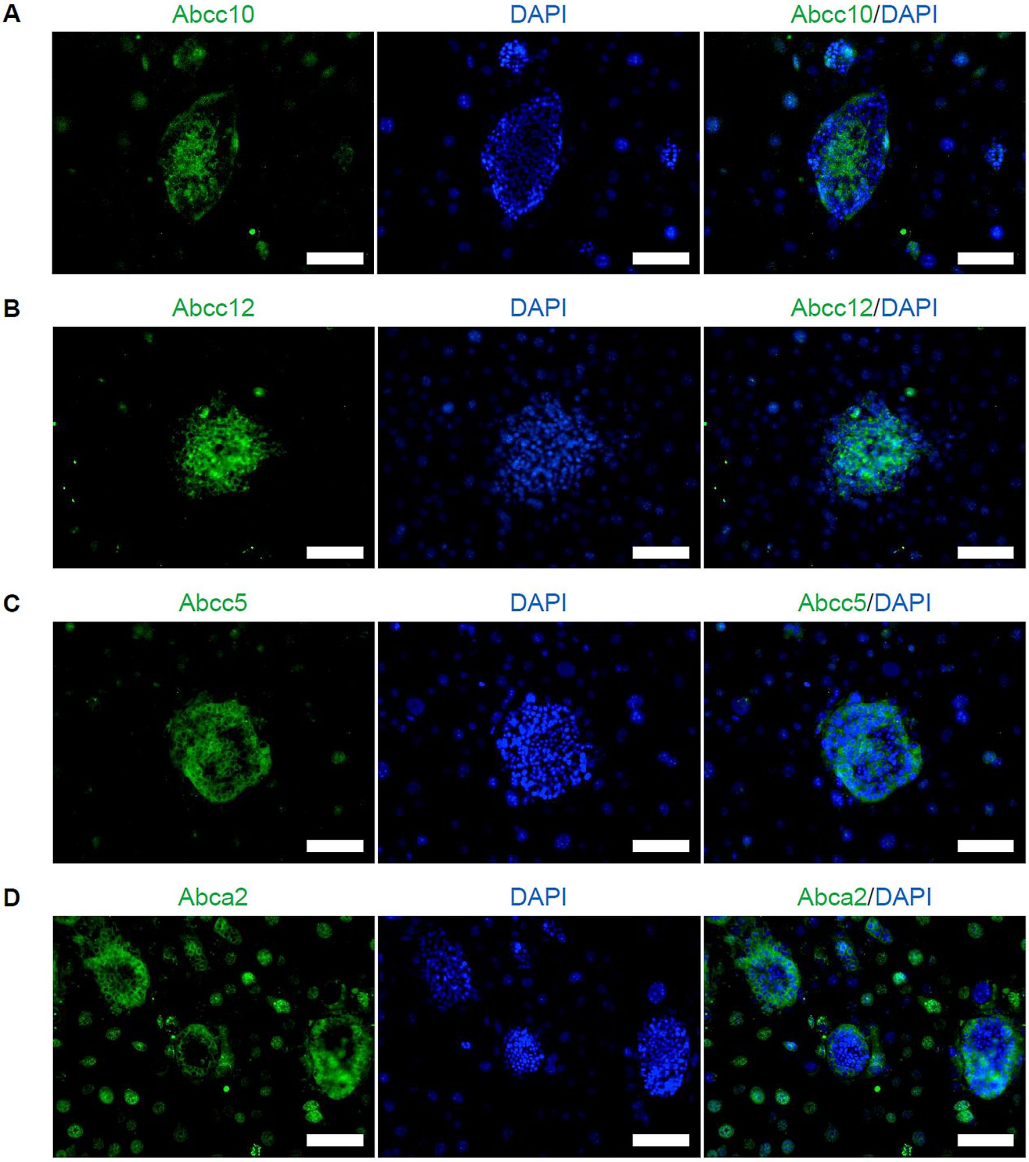
Immunohistochemistry. (**A**–**D**) miTS-P cells were fixed with 4% paraformaldehyde in PBS. After blocking with 20% AquaBlock for 30 min at room temperature, the cells were incubated overnight at 4 °C with the following antibodies: anti-Abcc10 antibody (**A**), anti-Abcc12 antibody (**B**), anti-Abcc5 antibody (**C**), or anti-Abca2 antibody (**D**). The cells were then incubated for 1 h at room temperature with FITC-conjugated secondary antibody. They were treated with mounting medium to detect the fluorescence emitted by DAPI.

Immunohistochemical analysis detected Abcc10 and Abcc12 expression by the central cells of miTS-P colonies (Fig. 6A,B). In contrast, Abcc5 was expressed in an irregularly mottled pattern by miTS-P colonies (Fig. 6C), and the border cells of miTS-P colonies expressed Abca2 (Fig. 6D). These data suggest that Abcc10 and Abcc12 participated in mediating the efflux of KP-1 from miTS-P cells.

## Discussion

Here we show that KP-1 distinguished between mES and miTS-P cells. Specifically, KP-1 stained mES cells to the same extent as hES/hiPS cells, while in striking contrast, KP-1 only stained border cells of miTS-P colonies. Our present findings extend our previous findings demonstrating that iPS cells are distinguished from iTS-P cells through their differential expression of Pdx1^25,28^. Compared with a study of human ES cells^18^, our study showed that mouse ES cells differentially express ABC transporters. Further, the mES and miTS-P cells exhibited different morphologies, with the mouse ES cells forming clusters while the miTS-P cells formed cobble-stone-like colonies, which formed a monolayer. Accordingly, we can exclude the possibility that KP-1 was unable to penetrate through larger colonies to reach the iTS-P cells.

ABCB1 and ABCG2 mediate the efflux of KP-1 from human cells, and the expression of each transporter is repressed in hES cells^36^. In contrast, our microarray and RT-qPCR data show that mES cells expressed Abcb1 and Abcg2, and previous studies show that ABCG2 is expressed at low levels in hES cells, while Abcg2 is expressed high-levels in mES cells^37^ Further, ABCG2 is expressed at high levels by hES cells^38^. Despite these conflicting data regarding the expression of ABCG2 in hES cells, we conclude from our present data and those of others^36–38^ that Abcb1 and Abcg2 are unlikely to cause the efflux of KP-1 from mouse cells. Further, our present microarray and RT-qPCR data lead us to conclude that Abcc5, Abcc10, Abcc12, and Abca2 may mediate the efflux of KP-1 from mouse cells.

Our present studies using the Abcc5 inhibitor sildenafil^33,34^, Abcc10 inhibitor CsA^18,35^, and siRNA of each ABC transporter show that Abcc10 and Abcc12 participate in the mechanism of efflux of KP-1 from miTS-P cells. The lipophilic anion transporter Abcc10^23,39–42^ mediates the transport of glucuronate conjugates such as E_2_17βG and GSH conjugates such as LTC_4_^41^. Further, Abcc10 may possess a bipartite substrate binding pocket that interacts with anionic and lipophilic ligands. The transport of E_2_17βG is competitively inhibited by organic anions such as LTC_4_, glycolithocholate 3-sulfate, and MK-571, as well as by lipophilic agents such as CsA^41^. Abcc12 was identified using a functional genomics approach and bioinformatics analysis^43,44^. The predicted amino acid sequence of Abcc12 exhibits the highest degree of similarity with Abcc5. Although the function of Abcc12 is unknown, it may differ from that of other family members^43,44^.

Our present findings that KP-1 stains only the border cells of colonies formed by miTS-P cells suggest that the ABC transporters were differentially expressed in central cells vs border cells of the colony. However, KP-1 stained most miTS-P cells on day 1 after cell passage, suggesting that the KP-1-negative central cells reversibly incorporated KP-1-positive cells after cell passage. The morphology of miTS-P colonies was cobblestone-like, and the central cells of the colony were contact-inhibited. Thus, the inhibition of cell division upon contacting a neighboring cell may prevent miTS-P cells from forming tumors. In contrast, border cells of the miTS-P colony and miTS-P cells on day 1 after cell passage were not contact-inhibited and continued to divide. Thus, contact inhibition may explain the ability of KP-1 to stain miTS-P cells.

In summary, we present compelling evidence that KP-1 distinguishes between mES cells and miTS-P cells, which serves as a useful tool to distinguish iPS and iTS-P cells that are generated using the same method. As iPS cells and iTS-P cells are generated by the same reprograming factors, KP-1 is useful for distinguishing between iPS cells and iTS-P cells. Our data further reveal differences between pluripotent stem cells and tissue-specific stem cells as well as the differences in expression of ABC transporters by human and mouse ES cells. KP-1 is therefore useful for selecting stem cells and for readily predicting the expression of patterns of ABC transporters.

## Methods

### Mice and cell culture

The University of the Ryukyus review committee approved experiments using mice. C57/BL6 mice (24-weeks-old) (CREA) provided the source of primary pancreatic tissue. Mouse pancreata were digested in 2 ml of cold M199 medium containing 2 mg/ml collagenase (Roche Boehringer Mannheim). The digested tissues were incubated in Dulbecco’s modified Eagle’s medium (DMEM; Invitrogen) containing 15% fetal bovine serum (FBS; BIO-WEST). NOD/scid mice (8-weeks-old) (CREA) were used to study teratoma formation. Mouse ES cells (ATCC) and iTS-P cells were maintained on feeder layers of mitomycin C-treated STO cells cultured in complete ES cell media containing 15% FBS (Millipore) as previously described^25–28^. ES cells were passaged every 4 days, and iTS-P cells were passaged every 4 to 5 days.

### Generation of iPS and iTS-P cells through replicon transfection

Generation of iTS-P cells was conducted as previously described^28^ using a Simplicom RNA Reprogramming Kit (Millipore). Pancreatic cells were added to T25 plates on day 0 and cultured to 90%–100% confluence on day 1. To minimize the interferon response, cells were first treated with 1 mL of Advanced DMEM containing 0.2 µg B18R protein 2 h before transfection. A mixture of 0.5 µg VEE-OKS-iG plus 0.5 µg B18R mRNAs was used to transfect cells in the presence of Lipofectamine 2000. After 3 h, transfection medium was replaced with Advanced DMEM containing 200 ng/mL B18R protein. On day 7, Advanced DMEM was replaced with ES culture medium. Puromycin (0.8 mg/ml) was added daily from days 2 to 10. Cells were passaged on STO feeder cells on day 10 and cultured in ES culture medium. Advanced DMEM containing 200 ng/mL B18R protein was supplied every day until iTS-P colonies were generated.

### Teratoma and tumorigenicity assay

miTS cells (1 × 10^6^–1 × 10^7^) were inoculated into each thigh of the NOD/scid mice. As a positive control, we transplanted 1 × 10^6^ mES cells into one thigh.

### KP-1 treatment

mES (RIKEN BRC Cell Bank, Tsukuba, Japan), miTS-P, and a mixture of miPS/miTS-P cells (2 × 10^5^ cells per well) were added to a six-well plate with mouse STO feeder cells. mES and miTS-P cells were similarly prepared without feeder cells. After cultured cells were incubated with 2 μM KP-1 (Goryo Kayaku Co. Ltd., Sapporo, Japan) for 3 h at 37 °C, cells were rinsed with PBS, and a fluorescence microscope was used to acquire images.

### Flow cytometric analysis of mES and miTS-P cells

After treating mES and miTS-P cells with KP-1, washing twice with ice-cold PBS, and dissociating them using 0.25% trypsin-EDTA, cells were counted using a Novocyte Flow Cytometer (ACEA Biosciences, Inc., San Diego, CA, USA) according to the manufacturer’s instructions.

### Microarray analysis

Microarray analysis was conducted as previously described^28^. Total RNA from ES or iTS-P cells was labeled with biotin. Samples were hybridized using a GeneChip 3´ IVT PLUS Reagent Kit (Affymetrix, Tokyo, Japan) and a CeneChip Hybridization, Wash, and Stain Kit (Affymetrix, Tokyo, Japan) according to the manufacturer’s protocol. Arrays were scanned using a GeneChip Scanner 3000 7G (Affymetrix, Tokyo, Japan), and were analyzed using Transcriptome Analysis Console 4.0 software (Thermo Fisher Scientific, Waltham, MA, USA).

### RT-qPCR analysis

RT-qPCR analysis was conducted as previously described^28^. Total RNA was extracted from cells using an RNeasy Mini Kit (QIAGEN, Tokyo, Japan). Spectrophotometrically quantified RNA (2.5 µg) was heated at 85 °C for 3 min and then reverse-transcribed in a 25-µl solution containing 200 units of Superscript II RNase H-RT (Thermo Fisher Scientific, Waltham, MA, USA), 50 ng random hexamers (Thermo Fisher Scientific, Waltham, MA, USA), 160 µmol/l dNTP, and 10 nmol/l dithiothreitol. The reaction was incubated for 10 min 25 °C, 60 min at 42 °C, and 10 min at 95 °C. Quantification of mRNA levels was performed using a TaqMan real-time PCR system according to the manufacturer’s instructions (Thermo Fisher Scientific). PCR was performed for 40 cycles, including 2 min at 50 °C and 10 min at 95 °C as initial steps. During each cycle, denaturation was performed for 15 s at 95 °C, and annealing and extension were performed for 1 min each at 60 °C. PCR was performed as well in a 20-µl of reaction mixture containing cDNAs synthesized from 1.11 ng of total RNA. For each sample, the level of mRNA was normalized to that of Gapdh. Primers for mouse Nanog (Mm02019550_s1), Sox2 (Mm03053810_s1), Oct3/4 (Mm03053917_g1), Lin28a (Mm00524077_m1), Nodal (Mm00443040_m1), Rex1 (Mm03053975_g1), Hnf1β (Mm00447459_m1), Hnf4α (Mm01247712_m1), Foxa2 (Mm01976556_s1), Sox17 (Mm00488363_m1), CD133 (Mm00477115_m1), Abcb1 (Mm00440736_m1), Abcg2 (Mm00496364_m1), Abcc5 (Mm01343626_m1), Abcc10 (Mm00467403_m1), Abcc12 (Mm01241948_m1), Abca2 (Mm00431553_m1) and Gapdh (Mm99999915_g1) are components of Assays-on-Demand Gene Expression Products (Thermo Fisher Scientific).

### ABC transporter inhibitors

After 5 days of passage, miTS-P were treated with 2 µM KP-1 for 3 h. Cells were rinsed with PBS before microscopy. The cells were then treated with 5 µM cyclosporin A (Tokyo Chemical Industry Co., Ltd., Tokyo, Japan) (an Abcc10 inhibitor) or 50 µM sildenafil (Selleckchem.com, USA) (an Abcc5 inhibitor) for 2 h, retreated with 2 µM KP-1 for 3 h and rinsed with PBS.

### RNA interference

After 5 days of passage, miTS-P cells were treated with 2 µM KP-1 for 3 h at 37 °C. Cells were rinsed with PBS before microscopy. The miTS-P cells were then transfected with 30 pmol siRNA of Abcc10, Abcc12, Abcc5, or Abca2 (Thermo Fisher Scientific, CA, USA) and cultured for 24 h. The miTS-P cells were then retreated with 1 µM KP-1 for 2 h at 37 °C and rinsed with PBS.

### Immunohistochemistry

miTS-P cells were fixed with 4% paraformaldehyde in PBS. After blocking with 20% AquaBlock (EastCoast Bio, North Berwick, ME, USA) for 30 min at room temperature, the cells were incubated overnight at 4 °C with a mouse anti-Abcc10 antibody (1:50; Abnova, Taipei, Taiwan), a rabbit anti-Abcc12 antibody (1:50; Bioss, MA , USA), a rabbit anti-Abcc5 antibody (1:50; Abcam, Tokyo, Japan), or a rabbit anti-Abca2 antibody (1:50; Abcam, Tokyo, Japan), and then for 1 h at room temperature with a donkey anti-mouse antibody (1:200; Thermo Fisher Scientific, CA, USA) or a goat anti-rabbit IgG H&L (1:200; Abcam, Tokyo, Japan). The miTS-P cells were treated with mounting medium to detect the fluorescence emitted by DAPI (Vector Laboratories, Peterborough, UK).

### Statistical analysis

Data are expressed as the mean ± SE. Two groups were compared using the Student *t* test. Differences between groups were considered significant if *p* < 0.05.

All methods were performed in accordance with the relevant guidelines and regulations.

## Supplementary information

Supplementary Information.

## Data Availability

All datasets are available from the corresponding author upon reasonable request.

## Abbreviations

iPS: Induced pluripotent stem
ES: Embryonic stem
iTS: Induced tissue-specific stem
iTS-P: ITS cells from the pancreas
KP-1: Kyoto probe
ATP-binding cassette transporters: ABC transporters
Sox: Sex-determining region Y-box
PCR: Polymerase chain reaction
RT-PCR: Reverse transcription PCR
DMEM: Dulbecco’s modified Eagle’s medium
FBS: Fetal bovine serum
MRP: Multidrug resistance-associated protein
