# Supplementary material for: Kyoto probe-1 reveals phenotypic differences between mouse ES cells and iTS-P cells
Source: Sci Rep. 2020 Oct 22;10:18084. doi: 10.1038/s41598-020-75016-6 (PMC7582910; doi:10.1038/s41598-020-75016-6)
Supplement: Supplementary file 1 — Supplementary Information. [file 41598_2020_75016_MOESM1_ESM.docx]

**Kyoto probe-1 reveals phenotypic** **differences between mouse ES cells and iTS-P cells**

Chika Miyagi-Shiohira,^1^ Issei Saitoh,^2^ Masami Watanabe,^3^ Hirofumi Noguchi,^1,*^

^1^Department of Regenerative Medicine, Graduate School of Medicine, University of the Ryukyus, Okinawa 903-0215, Japan

^2^Division of Pediatric Dentistry, Graduate School of Medical and Dental Science, Niigata University, Niigata 951-8514, Japan

^3^Department of Urology, Okayama University Graduate School of Medicine, Dentistry and Pharmaceutical Sciences, Okayama 700-8558, Japan

There was no Competing Financial Interests.

**Supplementary Figure Legends**

**Supplementary Figure 1. Characteristics of miTS-P cells using a synthetic self-replicating RNA.**

(A) Morphologies of miTS-P cells and mES cells, scale bars = 200 µm. (B) Numbers of colonies of miPS and miTS-P cells. The synthetic self-replicating RNA was used to transfect pancreatic tissue from mice aged 24 weeks, and the number of colonies was counted after 30–45 days. (C) RT-qPCR analysis of the expression of pluripotency marker genes Nanog, Sox2, Oct3/4, Lin28a, Nodal, and Rex1 and endodermal marker genes Hnf1β, 4α, Foxa2, Sox17, and CD133). **p*<0.05, ** *p*<0.01 (D) Teratoma formation/tumorigenicity assay. mES/miTS cells (1 × 10^6^ to 1 × 10^7^) were inoculated into each thigh of NOD/SCID mice. (E) Number of mice bearing teratomas.

**Supplementary Figure 2. Effects of siRNAs of ABC transporters.**

RT-qPCR analysis of Abcc5, Abcc10, Abcc12, and Abca2 expression. Mean ± SE. (n = 3). * *p*<0.05, ** *p*<0.01

**Supplementary Figure 1. Characteristics of miTS-P cells using a synthetic self-replicating RNA.**

**Supplementary Figure 2. Effects of siRNAs of ABC transporters.**
